# What intensity of exercise is most suitable for the elderly in China? A propensity score matching analysis

**DOI:** 10.1186/s12889-021-11407-2

**Published:** 2021-07-15

**Authors:** Xinlin Chen, Dai Su, Xinlan Chen, Yingchun Chen

**Affiliations:** 1 School of Medicine and Health Management, Tongji Medical College, Huazhong University of Science and Technology, Wuhan, 430030 China; 2 Research Centre for Rural Health Service, Key Research Institute of Humanities & Social Sciences of Hubei Provincial Department of Education, Wuhan, 430030 China; 3 Present address: Department of Health Management, School of Medicine and Health Management, Tongji Medical College, Huazhong University of Science and Technology, Wuhan, 430030 China

**Keywords:** Successful ageing, Intensity, Physical activity, The elderly

## Abstract

**Background:**

The strategy of successful ageing is an important means to deal with the challenges of the current ageing society. This paper aims to explore the effects of different intensities of physical activity on the successful ageing of the elderly.

**Methods:**

Our data were from wave 4 of the China Health and Retirement Longitudinal Survey (CHARLS), involving 9026 residents aged 60 years and older. The intensity of physical activity was divided into three levels: vigorous, moderate and mild. The concept of successful ageing adopted a four-dimensional model of life satisfaction added to the theoretical model of Rowe and Kahn’s. Propensity score matching (PSM) with controlling nine confounding factors were used to analyse the effects of different intensities of physical activity.

**Results:**

The percentage of successful ageing was 1.88% among all subjects. Among them, 30.26, 29.57 and 29.40% of the elderly often participated in vigorous, moderate and mild physical activity, respectively. The results of PSM showed that participation in moderate activity increased the probability of successful ageing of the elderly by 0.76–0.78% (*P* < 0.001), while participation in vigorous and mild physical activity had no significant effect on successful ageing (*P* > 0.05). Moderate physical activity had statistically significant effects on four components of successful aging, including major disease, physical function, life satisfaction, and social participation (*P* < 0.05).

**Conclusion:**

Moderate-intensity physical activity was most beneficial to the successful ageing of the elderly and should be promoted in the elderly population.

## Introduction

Ageing has become a major global challenge. By 2050, the population aged 60 and above will reach one-fifth of the total population [1]. Hence, to actively deal with the problem of population ageing and steadily improve a healthy life expectancy, the World Health Organization (WHO) strongly advocates the concept of healthy ageing and proposed the strategy of healthy ageing in 2015 with the aim of overcoming or minimizing the adverse consequences of population ageing through human intervention. Scholars have also extensively discussed healthy ageing. As early as the 1980s, American scholars Rowe and Kahn [2] proposed the classic definition of successful ageing, that is, successful ageing should simultaneously meet the three-dimensional theoretical model of avoiding disease and the functional loss, maintaining physical and cognitive functions, and actively participating in social activities. Since then, Gatz and Zarit [3] realized that subjective well-being was as important as objective measurement indicators such as health and cognition, and included it into the concept of successful ageing, forming a four-dimensional theoretical model consistent with the health concept of ‘good physiology, psychology and social adaptation’ proposed by the WHO. Successful ageing with healthy ageing, positive ageing, harmonious ageing, moderate ageing, secure ageing and other content as its content, should become an important magic weapon of ageing society governance [4].

Numerous studies have demonstrated that factors, such as demographic characteristics [5–7] health-related behaviours [8–10] and family and social support [11, 12] may contribute to successful ageing. In recent years, active participation in physical activity has also been highlighted as an important factor in promoting successful ageing, including reducing the risk of all-cause death [13], chronic disease [14], functional loss [15], reducing anxiety and depression [16, 17] and improving cognition [18]. Some of the health benefits of exercise for older adults are related to its intensity [19]. However, some scholars have debated on what level of intensity of exercise is most appropriate for health in the elderly. Swain DP et al. [20] and Gormley Se et al. [21] found that compared with moderate-intensity exercise, high-intensity exercise could improve aerobic capacity and cardiac protection to a greater extent. Liu Xiaodi et al. [22] pointed out that high-intensity exercise could improve the memory of the elderly, while moderate-intensity exercise had no significant effect. On the contrary, Wannamethee SG et al. [23] Woodcock I et al. [24] and Cao Ruofan et al. [25] supported the protective effect of moderate- and mild-intensity exercise on the physical and mental health of the elderly and believed that vigorous exercise did not have more benefits. At the same time, previous studies have focused more on the effects of exercise on a single disease or function, and few studies considered the effects of exercise on successful ageing, which is a more comprehensive concept of health. Therefore, we hope to explore the relationship between the different intensities of physical activity and successful ageing, and propose exercise prescriptions to help the elderly successfully ageing. This study hypothesised that moderate intensity of physical activity would be most beneficial for successful aging in older adults, with higher risk associated with too high intensity and insignificant benefits with too low intensity.

## Methods

### Data and sampling

The data used in this study were collected from wave 4 of the China Health and Retirement Longitudinal Survey (CHARLS), which is available at http://charls.pku.edu.cn. The database is a national survey among Chinese adults over the age of 45. Using a stratified (calculated by per capita GDP of urban and rural counties) multi-stage (county/district-village/community-household) PPS (probability proportional to scale) sampling strategy, the survey was conducted in 150 counties and 450 communities (villages) of 28 provinces (autonomous regions and municipalities) in 2011, 2013, 2015 and 2018 [26]. We collected 19,816 observation data from the 2018 CHARLS database. We selected the elderly over 60 years old as the research object based on the purpose of the study. After eliminating the missing values of successful ageing, physical activity and some basic characteristics, 9026 effective samples were finally obtained.

### Measurements

#### Successful ageing

We adopted the four-dimensional conceptual model of successful ageing and defined successful ageing as the following components: (1) no major disease, (2) no disability, (3) high cognitive function, (4) no loss of physical function, (5) active social participation, and (6) high life satisfaction.

Major disease:

Chronic diseases are the biggest threat to the health of older people. We considered six chronic diseases that currently cause premature death among the elderly in China: hypertension, cancer or malignant tumour, chronic lung diseases such as chronic bronchitis and emphysema, heart disease, stroke and memory-related diseases [27]. The absence of any of the above diseases was considered to be free of major diseases.

#### Disability

Participants were asked if they had a disability, including physical disability, brain damage/intellectual disability, vision problem, hearing problem and a speech impediment. If none, they were deemed to have no disability.

#### Physical function

Physical function status was measured by the Activity of Daily Living Scale (ADL) [28], including six options, namely, using the toilet, eating, dressing, getting into or out of bed, walking and bathing. The four-level score was adopted: ‘Have no difficulty =1’, ‘Have difficulty but can still do it =2’, ‘Have difficulty and need help =3’ and ‘Cannot do it =4’. Any score greater than 1 was recorded as a loss of physical function.

#### Cognitive function

Mini-Mental State Examination scale (MMSE) [29] was used to evaluate. The MMSE includes orientation, memory, attention and numeracy, recall and language skills, with a total score of 30. A score of < 17 for illiteracy, < 20 for elementary school and < 24 for secondary school or above were defined as impaired cognitive function.

#### Social participation

Active social participation was defined as having participated in at least one of the following social activities during the past month: (1) Interacted with friends; (2) Played Mah-jong, played chess or played cards; (3) Provided help to family, friends or neighbours who do not live with you; (4) Went to a club; (5) Took part in a community-related organization; (6) Done voluntary or charity work; (7) Cared for a sick or disabled adult who does not live with you; (8) Attended an educational or training course; (9) Stock investment; (10) Used the Internet and (11) Other.

#### Life satisfaction

In the CHARLS questionnaire, a single-choice question was set: ‘Please think about your life-as-a-whole. How satisfied are you with it?’ The answers were ‘completely satisfied’, ‘very satisfied’, ‘somewhat satisfied’, ‘not very satisfied’ and ‘not at all satisfied’. The first three items were assigned a score of 1, which meant high life satisfaction. If the answer was the last two items, the score was 0, which meant low life satisfaction.

#### Physical activity

According to the International Physical Activity Questionnaire - Short Form (IPAQ-SF) [30], the physical activity of the elderly was divided into three intensity levels: vigorous, moderate and mild. Among them, vigorous activities could cause shortness of breath, such as carrying heavy stuff, digging, hoeing, aerobic workout, bicycling at a fast speed and riding a cargo bike/motorcycle; moderate activities could make people breathe faster than usual, such as carrying light stuff, bicycling at a normal speed, mopping, Tai-Chi and speed walking. One example of mild activities was walking, which included walking from one place to another place at a workplace or home, and taking a walk for leisure, sports, exercise or entertainment. For each level of activity, the respondents were asked, “Do you usually take this type of activity for at least 10 minutes every week?” If the answer was ‘yes’, it was considered that they often participated in physical activities of this intensity. If the subjects answered ‘yes’ to multiple questions, they were classified according to the activity with the highest intensity. For example, if the answer to vigorous intensity and mild intensity activities was ‘yes’, the subjects were classified as frequently participating in vigorous-intensity physical activities.

#### Covariates

The potential confounding factors between physical activity and successful ageing outcomes and the predictors of successful ageing outcomes were selected as covariates, including demographic characteristics (gender [31], age [32], marital status [33], education level [34] and residential area [35]), health behaviour (smoking [36], drinking [36] and sleep duration per day [37]) and medical insurance [35], a total of nine variables (Table 1).

**Table 1:** Covariate measurement and attributes

Variable	Mean (SD) or frequency (%)
Gender (Female)	51.09
Age	66.16 (4.02)
Marital status	
Married and live with a spouse	80.68
Married but not living with spouse temporarily for reasons such as work	4.17
Separated, no longer live together as a couple anymore	0.26
Divorced	0.85
Widowed	13.45
Never married	0.56
Cohabitation	0.04
Education background	
No formal education (illiterate)	26.80
Did not finish primary school	24.63
Sishu/Home school/Elementary school	21.43
Middle school	17.29
High school	6.60
Vocational school	2.09
Two−/Three-Year College/Associate degree	0.77
Four-Year College/Bachelor’s degree /Master’s degree/Doctoral degree/Ph.D.	0.39
Smoking (Yes)	47.58
Drinking	
No	69.98
Drink but less than once a month	7.38
Drink more than once a month	22.64
Sleep duration per day	6.19 (2.04)
Residential area	
Central of City/Town	19.55
Urban-Rural Integration Zone	7.25
Rural	72.83
Special Zone	0.37
Insurance (Yes)	96.81

### Statistics analysis

Categorical variables were displayed with percentage, while numerical variables were displayed with mean and standard deviation. Propensity Score Matching (PSM) proposed by Rosenbaum and Rubin (1983) [38] was used in this study to evaluate the effects. The main advantage of this method is that after adding covariates to pair the treatment and control groups, the results are similar to those of natural experiments, which makes the sample distribution of the treatment and control groups close to random distribution to ensure that the distribution of the experimental results and the treatment variables are independent of each other, meet the condition independence hypothesis and reduce the selectivity error [39]. The elderly who engaged in vigorous-, moderate- and mild-intensity physical activity every week were the intervention groups, and the elderly without certain physical activity were the corresponding control group, with the successful ageing status as the result of different intensity physical activities. We built the Logit model to predict the probability of the elderly participating in physical activity, and conducted matching based on the propensity score to estimate the average treatment effect of treated (ATT).

1 $$ \mathrm{P}\ \left(\mathrm{Xi}\right)=\mathrm{P}\ \left(\mathrm{Di}=1|\mathrm{X}=\mathrm{Xi}\right) $$

2 $$ \mathrm{ATT}=\mathrm{E}\ \left[\mathrm{Yi}\ (1)\ |\mathrm{P}\ \left(\mathrm{Xi}\right),\mathrm{Di}=1\right]-\mathrm{E}\ \left[\mathrm{Yi}\ (0)\ |\mathrm{P}\ \left(\mathrm{Xi}\right),\mathrm{Di}=1\right] $$

Di is the indicator variable. When the research object was engaged in a certain intensity of physical activity, Di is 1, otherwise, it is 0; Xi is a series of characteristic variables affected by whether the research object participated in physical activity; Yi (1) and Yi (0) respectively indicated the successful ageing status of the elderly in the intervention and control groups. To ensure robustness of the estimation results, we used three different methods for sample matching, including the nearest neighbour matching within the calliper, radius matching and kernel matching. The estimation results could be regarded as robust if the final results were similar. According to the principle of ε ≤ 0.25σ̂_pscore, we set the calliper range of nearest neighbour matching and radius matching as 0.001. Kernel matching used a quadratic kernel, and the bandwidth was set to 0.06. All data compilation and statistical analysis were performed using STATA 15.0.

## Results

### Exercise and successful ageing status of the elderly

First, a descriptive analysis was carried out on the physical activity and successful ageing of the elderly, and a one-way ANOVA was used to compare the differences in successful aging among the three groups performing high-intensity, moderate-intensity, and mild-intensity exercise. Among the research subjects, the percentage of successful ageing was 1.88%. 30.26, 29.57 and 29.40% of the elderly often participated in vigorous, moderate and mild intensity physical activities, respectively. There was no statistically significant difference in successful aging among the three exercise intensity groups (*P* > 0.05, Table 2).

**Table 2:** Participation in physical activities of different intensities and successful aging of the elderly

		Total n (%)	Successful aging	Unsuccessful aging	*P-value*
Vigorous physical activity		2731 (30.26)	51 (1.87)	2680 (98.23)	0.27
	No	6295 (69.74)	119 (1.89)	6176 (98.11)	
Moderate physical activity	Yes	2669 (29.57)	64 (2.40)	2605 (97.60)	
	No	6357 (70.43)	106 (1.67)	6251 (98.33)	
Mild physical activity	Yes	2654 (29.40)	49 (1.85)	2605 (98.15)	
	No	6372 (70.60)	121 (1.90)	6251 (98.10)	
Total		9026	170 (1.88)	8856 (98.12)	

### Propensity score

First, a prediction model was established using Logit regression to calculate the propensity of the elderly in the sample to engage in various levels of physical activity. Table 3 showed that the gender, education level, residential area and drinking had statistical significance in whether to participate in vigorous-intensity physical activity. Gender, residential area and health insurance had statistical significance in whether to participate in moderate-intensity physical activity. Meanwhile, education background, drinking and sleeping duration per day had statistical significance in whether to participate in mild-intensity physical activity (*P* < 0.05).

**Table 3:** Logit model estimation results of factors influencing physical activities of the elderly

	Vigorous physical activity	Moderate physical activity	Mild physical activity
Gender	3.08 (0.002)	−2.04 (0.041)	−0.45 (0.650)
Age	−0.36 (0.722)	− 0.87 (0.384)	0.98 (0.328)
Marital status	1.39 (0.165)	−0.73 (0.466)	0.40 (0.686)
Education background	−4.04(< 0.001)	0.82 (0.414)	2.92 (0.003)
Residential area	5.32(< 0.001)	−3.82(< 0.001)	− 1.43 (0.153)
Smoking	0.03 (0.973)	− 1.94 (0.052)	1.28 (0.201)
Drinking	9.83(< 0.001)	−1.64 (0.100)	− 3.04 (0.002)
Sleeping duration per day	−1.57 (0.116)	0.30 (0.765)	2.17 (0.030)
Insurance	−0.29 (0.773)	2.68 (0.007)	−1.04 (0.161)
Constant	−3.70(< 0.001)	−2.18 (0.029)	− 3.68(< 0.001)
LR chi2	178.22	18.79	33.48
Prob > chi2	< 0.001	42.81	< 0.001
Pseudo-R2	0.0161	0.0039	0.0031
Observations	9026		

### Balance test

The balance assumption needs to be verified to ensure the quality of matching and the reliability of the estimated results. The balance test of matching variables is usually used to investigate whether the individual differences of each matching variable had been significantly reduced after matching, and the most commonly used index is the mean of deviation. Table 4 reported the balance test results of the characteristic variable matching between the intervention and the control groups, taking the nearest neighbour matching as an example. The results showed that the standardized deviations of most of the matching variables in the treatment and control groups were significantly reduced, and the standardized deviations were controlled within 5%. T-test results indicated that no significant difference was observed between the two elderly sample groups after matching, indicating that the matching effect was good.

**Table 4:** Balance test results

Variables	U/M	Vigorous physical activity		Moderate physical activity		Mild physical activity	
		%bias	*P*-value	%bias	*P*-value	%bias	*P*-value
Gender	U	4.2	0.070	−4.4	0.058	1.1	0.636
	M	1.8	0.509	1.2	0.659	0.2	0.942
Age	U	1.7	0.462	−3.0	0.201	1.7	0.471
	M	3.2	0.230	2.1	0.431	1.2	0.651
Marital status	U	2.9	0.201	−2.0	0.396	1.1	0.647
	M	2.4	0.372	1.9	0.479	2.4	0.387
Education background	U	−13.3	0.000	4.7	0.041	8.2	< 0.001
	M	1.2	0.650	1.4	0.606	1.0	0.723
Residential area	U	17.4	< 0.001	−10.6	< 0.001	−6.3	0.006
	M	0.5	0.830	−2.8	0.319	−3.2	0.248
Smoking	U	3.4	0.139	−5.2	0.025	2.0	0.387
	M	1.0	0.717	1.2	0.663	−0.5	0.869
Drinking	U	22.5	< 0.001	−4.5	0.055	−6.7	0.004
	M	−1.1	0.705	3.5	0.196	3.0	0.262
Sleeping duration per day	U	−2.9	0.221	0.6	0.809	4.8	0.035
	M	−2.7	0.305	−0.1	0.984	−0.5	0.853
Insurance	U	−1.0	0.650	6.8	0.005	−3.0	0.191
	M	−1.2	0.660	0.5	0.833	−0.3	0.905

*U* unmatched, *M* matched

### Matching results

The results of PSM model analysis showed that participation in moderate physical activity increased the probability of successful ageing among the elderly by 0.76–0.78% (*P* < 0.001), while participation in vigorous and mild physical activities had no significant effect on successful ageing (*P* > 0.05). The results of the three matching methods were roughly the same, indicating that the results had good robustness (Table 5).

**Table 5:** Effects of different intensity of physical activity on successful aging of the elderly

	Vigorous activity	Moderate activity	Mild activity
Nearest neighbor matching within caliper	−0.0005(0.0035)	0.0078*(0. 0037)	−0.0003 (0. 0036)
radius matching	0.0013(0.0032)	0.0076*(0. 0034)	−0.0004(0. 0032)
Kernel matching	0.0003(0.0032)	0. 0076*(0. 0034)	−0. 0008(0. 0031)

Note: *P < 0.01

### Parameters of the successful aging components

Table 6 showed the parameters of the successful aging components by the nearest neighbour matching within the calliper. Participation in moderate-intensity activity had a statistically significant effect on major disease, physical function, social activity participation, and life satisfaction in older adults (*P* < 0.05), whereas participation in mild-intensity activity had no significant effect on all aspects of successful aging (*P* > 0.05). Participation in vigorous physical activity increased the probability of good physical function among the elderly by 8.71% (*P* < 0.001), which was greater than the effect of moderate-intensity activity (5.77%).

**Table 6:** Effects of different intensity of physical activity on the components of successful aging of the elderly

	Vigorous activity	Moderate activity	Mild activity
Major disease	−0.0134 (0.0139)	0.0300*(0.0138)	0.0062 (0.0138)
Disability	0.0142 (0.0139)	−0.0134 (0.0138)	−0.0161 (0.0139)
High cognitive function	−0.0222 (0.0138)	0.0068 (0.0137)	0.0117 (0.0137)
Physical function	0.0871*(0.0147)	0.0577*(0.0144)	−0.0240 (0.0145)
Social participation	0.0225 (0.0140)	0.1032*(0.0138)	−0.0171 (0.0140)
Life satisfaction	−0.0088 (0.0096)	0.0251*(0.0094)	−0.0166 (0.0096)

Note: *P < 0.05

## Discussion

This study used wave 4 data of CHARLS (a nationally representative sample of older Chinese) to explore the causal effects of different intensities of physical exercise on successful ageing. We adopted the PSM method to solve the bias estimation caused by non-randomized studies and control various confounding factors.

Compared with the results of studies on large samples in other countries, the level of successful ageing in China was very low. One of the reasons may be because of the different setting of ageing standards in various studies. Hank et al. (2011) [40] reported that the average probability of successful ageing in 15 European countries was 8.5%, which varied greatly in different countries, with the highest probability of 21.1% in Denmark and only 1.6% in Poland. Sara J McLaughlin et al. (2009) [41] reported a decline in the percentage of successful ageing in the United States from 11.9 to 10.9% during 1998–2004. However, neither study considered the life satisfaction dimension. Qiush Feng et al. (2015) [42] found that the rate of successful ageing among the elderly in South Korea was 25.2% when a less restrictive disease assessment criteria (four diseases) than in this study is used. According to the survey by Ana C Canedo et al. (2018) [43], the proportion of successful ageing in Brazil was 25%, excluding active participation and life satisfaction. Nevertheless, it cannot be denied that the health of the elderly in China is facing serious challenges.

Chinese elderly people had high enthusiasm to participate in sports, with 89.23% of the elderly often participating in physical activities of mild intensity or above (Table 2). The participation rate of each intensity activity was similar. The results of the PSM analysis showed that moderate physical activity had a significant positive effect on successful ageing, while vigorous and mild physical activity had no significant effect. Therefore, the optimal intensity of exercise for the elderly should be moderate intensity, which was consistent with the results of Jansson E et al. (2015) [44]. Physiologically, exercise can partially reverse the effect of the ageing process on the physiological function and maintain the functional reserve of the elderly [45], and its mechanisms include improving vascular endothelial function [46], increasing maximal oxygen uptake [46], increasing muscle strength [47, 48] and maintaining bone mass [49]. Our analysis results showed that there were more significant improvements at higher levels of exercise while exercise below the minimum threshold did not produce enough challenge to the body (Table 6), consistent with the findings of Garber CE et al. (2011) [50] and Duncan GE et al. (2005) [51] However, the elderly is more vulnerable, strenuous exercise may lead to sudden death or myocardial infarction [52] and cause musculoskeletal injuries [53]. Thus, moderate-intensity exercise could best bring maximum utility with minimal risk. From a psychological point of view, exercise can improve the elderly’s self-efficacy and reduce negative emotions [54]. The relationship between exercise intensity and emotional response can be modelled as an inverted U curve [55, 56], which means that moderate exercise corresponds to optimal emotional change. Low levels of exercise were less likely to yield emotional benefits. The pleasure gained from exercise decreases when the intensity of exercise is high, because older people sometimes forgo leisurely exercise for entertainment in the interest of physical fitness by exerting a higher intensity on themselves, and thus experience less pleasure [57]. From the perspective of social participation, the active participation of the elderly in physical activities is conducive to the improvement of the social network [58]. Older people tend to become more optimistic and more active in daily activities through exercise. In addition, most of the physical activities of the elderly are accumulated through going out, which is conducive to meeting the needs of interpersonal communication [59]. As for the relationship between different exercise intensities and social participation, not enough empirical studies have been conducted in this area to enable us to analyse and draw conclusions. In conclusion, moderate physical activity is most conducive to successful ageing.

### Limitations

This study has several limitations. First, the amount of exercise includes two aspects: intensity and frequency. This study only discusses the intensity of exercise. Thus, the appropriate exercise frequency still requires further study. Second, the assessment of exercise intensity is derived from the subjective judgment of each research object rather than the objective measurement of pulse, blood pressure and respiratory rate, and thus, some errors may still be observed. Third, the current ageing standards are not clear, and the results generated by different standards may be biased. This study adopts the classic concept of successful ageing and combines it with the current epidemic status of diseases to obtain the most accurate measurement of successful ageing as possible.

## Conclusion

Exercise in older persons should be encouraged while minimizing the risks and ensuring safe participation to maximize its benefits. Our study confirmed that only moderate-intensity physical activity was significant for the successful ageing of the elderly, of which nearly one-third of the elderly participate. Therefore, the elderly should be popularized the correct concept of sports and encouraged to participate in moderate-intensity physical exercise.

## Data Availability

The CHARLS dataset used in this study was publicly available at http://charls.pku.edu.cn.
